# Lineage plasticity and treatment resistance in prostate cancer: the intersection of genetics, epigenetics, and evolution

**DOI:** 10.3389/fendo.2023.1191311

**Published:** 2023-06-30

**Authors:** Jarrell Imamura, Shinjini Ganguly, Andrew Muskara, Ross S. Liao, Jane K. Nguyen, Christopher Weight, Christopher E. Wee, Shilpa Gupta, Omar Y. Mian

**Affiliations:** ^1^ Taussig Cancer Institute, Cleveland Clinic, Cleveland, OH, United States; ^2^ Glickman Urologic Institute, Cleveland Clinic, Cleveland, OH, United States; ^3^ Department of Pathology, Robert J. Tomsich Pathology and Laboratory Medicine Institute, Cleveland Clinic, Cleveland, OH, United States

**Keywords:** neuroendocrine prostate cancer, lineage plasticity, epigenetic dysregulation, hormone therapy resistance, radioresistance

## Abstract

Androgen deprivation therapy is a cornerstone of treatment for advanced prostate cancer, and the development of castrate-resistant prostate cancer (CRPC) is the primary cause of prostate cancer-related mortality. While CRPC typically develops through a gain in androgen receptor (AR) signaling, a subset of CRPC will lose reliance on the AR. This process involves genetic, epigenetic, and hormonal changes that promote cellular plasticity, leading to AR-indifferent disease, with neuroendocrine prostate cancer (NEPC) being the quintessential example. NEPC is enriched following treatment with second-generation anti-androgens and exhibits resistance to endocrine therapy. Loss of *RB1*, *TP53*, and *PTEN* expression and *MYCN* and *AURKA* amplification appear to be key drivers for NEPC differentiation. Epigenetic modifications also play an important role in the transition to a neuroendocrine phenotype. DNA methylation of specific gene promoters can regulate lineage commitment and differentiation. Histone methylation can suppress AR expression and promote neuroendocrine-specific gene expression. Emerging data suggest that EZH2 is a key regulator of this epigenetic rewiring. Several mechanisms drive AR-dependent castration resistance, notably AR splice variant expression, expression of the adrenal-permissive 3βHSD1 allele, and glucocorticoid receptor expression. Aberrant epigenetic regulation also promotes radioresistance by altering the expression of DNA repair- and cell cycle-related genes. Novel therapies are currently being developed to target these diverse genetic, epigenetic, and hormonal mechanisms promoting lineage plasticity-driven NEPC.

## 1 Introduction

In American men, prostate cancer (PCa) is among the most common malignancies and is projected to become the second leading cause of cancer related death in 2023, accounting for an estimated 29% of total new cases and 11% of total cancer related deaths (1). A rare but particularly lethal subtype is neuroendocrine PCa (NEPC), with a median survival of 7 months following diagnosis (2). The prevailing hypothesis behind the development of NEPC is the transdifferentiation of an adenocarcinoma to a neuroendocrine (NE) lineage (3). Generally, this NE phenotype is positive for markers of other high-grade NE carcinomas, such as chromogranin A(CHGA), neuron-specific enolase (NSE), synaptophysin (SYP), and CD56, and negative for luminal prostate differentiation markers (4). The transition to a NE phenotype is strongly associated with lineage plasticity. Lineage plasticity refers to the ability of cells to transition from one committed development program to another, allowing for multiple, varying phenotypes to arise from a singular genotype in response to environmental stimuli (5). This property is often maintained in cancer cells and is exploited to promote opportunistic adaptation and progression with plastic phenotypes being enriched in advanced stages of cancer. Therefore, lineage plasticity allows cancer cells to derive therapy resistance by reprogramming to a phenotype that is indifferent towards cellular pathways being targeted by therapy (3). Indeed, the lineage transition to a NE subtype is one mechanism by which PCa evades androgen receptor (AR)-targeting treatment by shifting its cellular phenotype from an AR-dependent adenocarcinoma to an AR-indifferent NE or small-cell carcinoma.

Unique to PCa is its reliance on the AR to regulate tumor progression via transcriptional regulation of AR targeted genes (6) and the activation of AR-targeted signaling pathways (7). Targeting the AR driven signaling cascade has proven effective in treating PCa (8) and has led to the emergence of androgen deprivation therapy (ADT) as a mainstay of treatment for locally advanced and metastatic PCa (9, 10). However, disease control using ADT alone is limited, and resistance is common, typically occurring after an average of 2-3 years of treatment (11). Recurrent tumors, known as castration-resistant prostate cancer (CRPC), are typically more aggressive, proliferating despite castrate levels of androgen. The emergence of a CRPC subtype is thought to be driven via androgen-dependent and androgen-independent mechanisms. The majority of CRPCs retain a degree of dependency on the AR and androgen signaling but escape ADT by a variety of mechanisms, including increased AR expression, expression of mutant AR variants, AR splice variant (AR-Vs) expression, and intratumoral steroid hormone synthesis (8). However, increased usage of AR-targeting treatments has been associated with an increased recognition of androgen-independent CRPC variants, with NEPC historically being the prototypic example of AR (-) CRPC (12, 13). Additionally, the AR’s role as a differentiation factor suggests its loss may play a significant role in lineage plasticity between CRPC subtypes (14), although numerous other factors have been implicated in driving PCa lineage plasticity. Notably, the development of CRPC may also promote resistance to radiotherapy (RT) as molecular pathways governing the transition to CRPC are often implicated in radioresistance (15–18). In this review, we discuss the genetic and epigenetic drivers of PCa lineage plasticity and link emergent phenotypes to treatment resistance. Specifically, we focus on the emergence of NEPC following ADT and the implications NE and other AR-indifferent phenotypes have on ADT and RT resistance.

## 2 Molecular mechanisms driving treatment emergent lineage plasticity

### 2.1 AR

For decades, androgens have been implicated in driving PCa development and progression through their interactions with the AR, a ligand-dependent nuclear transcription factor (6). With the androgen axis playing such a significant role in tumor progression, treatment plans frequently involve suppressing the androgen signaling axis via surgical or medical castration (19, 20). Several categories of modern ADT drugs can be used to accomplish medical castration, including luteinizing hormone-releasing hormone agonists/antagonists, AR antagonists, and androgen synthesis inhibitors (19, 20). First-generation AR antagonists were introduced in the late 1980s and include flutamide, nilutamide, and bicalutamide (21–23). These drugs are non-steroidal anti-androgens that act as selective competitive antagonists (22). Initial treatment with first-generation AR agonists is effective at reducing tumor burden and decreasing serum prostate-specific antigen levels; however, within a few years, many patients receiving ADT manifest early signs of biochemical progression, signaling the emergence of CRPC (11). Despite its classification, the majority of CRPC is still AR-dependent. As such, treatment with second-generation anti-androgens, such as enzalutamide, apalutamide, and darolutamide, have demonstrated clinical benefit, though typically for a limited duration. Treatment of CRPC with enzalutamide increased time to progression by 8.3 months in the AFFIRM trial, 11.2 months in the PREVAIL trial, and 19.4 months in the TERRAIN trial (21). Further progression following second-generation anti-androgen treatment can be mediated by the activation of the AR through secondary alterations to the AR gene, AR bypass or crosstalk mechanisms, or other means. However, increasing evidence suggests that a subset of CRPC tumors evade potent hormone therapy through a loss rather than a gain in AR function (24). These androgen-indifferent tumors become less dependent on AR signaling, a process associated with lineage plasticity, loss of luminal markers, and acquisition of NE features (24). Moreover, this AR-independent state is defined by a reduced reliance on the AR signaling pathway to promote tumor progression and the activation of other factors that promote cell survival such as MYCN, AURKA, ONECUT2, and BRN2 (3, 25, 26). Recently, it has been clarified that lineage plasticity is not a binary switch but occurs on a continuum with identifiable, preexisting clonal subsets. These subsets include AR activity-low tumors that exhibit decreased AR signaling despite continued AR expression, amphicrine tumors that express an active AR and NE program, double negative tumors that lack both AR and NE gene expression, and finally, NEPC tumors that lack AR expression and express a NE phenotype (12). Among these NE-negative subsets, NEPC is the dominant form, accounting for as much as 10-20% of CRPC (27, 28). Furthermore, NEPC incidence is expected to increase with widespread use of modern androgen receptor pathway inhibitors (ARPIs) (28).

### 2.2 RB1, TP53, and PTEN

**Figure 1:**
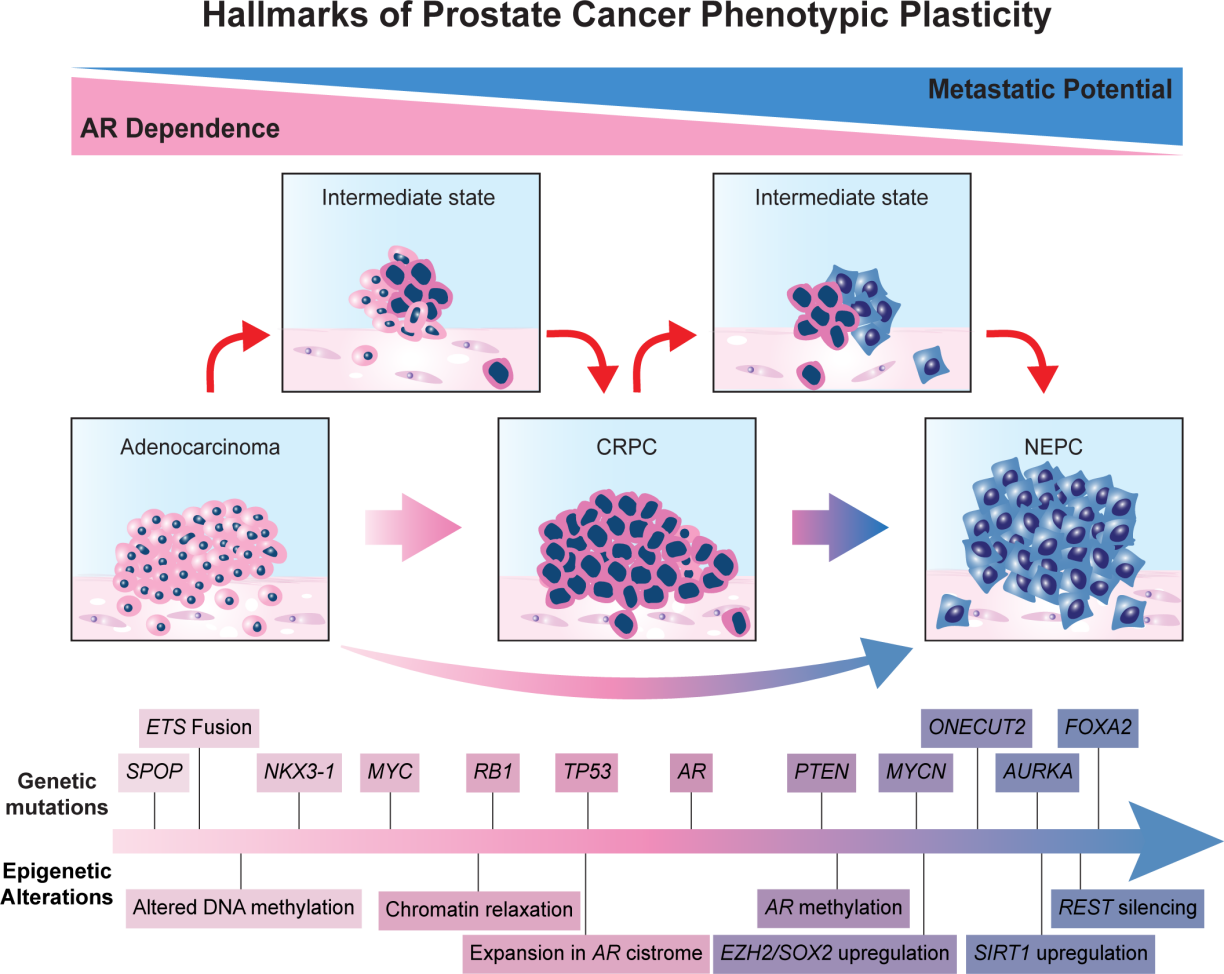
Hallmarks of prostate cancer phenotypic plasticity.

Recent genomic analysis has revealed several alterations enriched in NEPC. Mutations in the tumor suppressor genes *RB1*, *TP53*, and *PTEN* are individually among the most common genetic alterations in PCa. However, the concomitant loss of more than one of these critical factors is enriched in the NE subtype (4, 29, 30). For instance, concurrent *RB1* and *TP53* loss was present in 53.3% of NE-CRPC biopsies, compared with 13.7% of castration-resistant adenocarcinoma (4). Emerging data places special emphasis on the loss of RB in promoting phenotypic plasticity towards NEPC (31). Interestingly, aggressive NE tumors from other organs also exhibit alterations in these tumor suppressor genes (32). Investigation of a combined knockdown of *RB1* and *TP53* in *PTEN*-null LNCaP cells resulted in reduced AR expression, enzalutamide resistance, and activation of a NEPC program. Additionally, *SOX2* knockdown stopped the lineage plasticity program induced by *RB1*/*TP53* loss, signaling the importance of lineage plasticity related transcription factors in driving the NEPC transition (33). *In vivo* comparison between *PTEN* loss (SKO), *PTEN* and *RB1* loss (DKO), and *PTEN*, *RB1*, and *TP53* loss (TKO) has demonstrated lower AR expression and activation of NEPC programming in the DKO and TKO groups. Notably, the DKO and TKO tumors exhibited increased expression of *SOX2*, a key downstream effector for NEPC lineage plasticity (34). Moreover, loss of *RB1*, *TP53*, and *PTEN* reportedly induce NE transdifferentiation by upregulating NE program-related transcription factors, such as SOX2, SOX11, and EZH2 (33–35). Evidently, *RB1*, *TP53*, and *PTEN* loss are important factors in the transition to NEPC; however, evidence suggests that these genetic alterations alone are insufficient to promote lineage plasticity. For instance, there are numerous instances of NEPC tumors not exhibiting these mutations, and non-NE CRPC tumors have been observed exhibiting these genetic alterations (4, 36). Moreover, *RB1*/*TP53* knockouts of *PTEN*-deficient LNCaP cells resulted in decreased AR transcriptional activity but did not promote an increase of NEPC gene expression. This suggests necessity without sufficiency and that other reprogramming factors absent from these analyses may be required to induce a NE phenotype in PCa (36). These and other alterations that can be considered hallmarks of phenotypic lineage plasticity in prostate cancer are detailed schematically in **Figure 1**.

### 2.3 MYCN and AURKA

The phenotypic transition that leads to NEPC is also frequently associated with amplification of *MYCN* and *AURKA*, with concurrent amplification of these two genes occurring in 40% of NEPC compared to 5% of prostate adenocarcinoma (37). Preclinical models have found *MYCN* to be a key driver of NEPC phenotypes (37–39). *MYCN* overexpression in models with *PTEN* loss or *AKT* overexpression resulted in tumors with low *AR* expression and signaling, upregulation of NE markers, and resistance to AR targeting therapies (38, 39). Moreover, these tumors, along with *MYCN* overexpressing cell lines, demonstrated enriched expression of embryonic stem cell and active epithelial-mesenchymal transition (EMT) program gene sets (38, 39). Additionally, MYCN can be stabilized by dimerization with its allosteric protein partner, AURKA (40). Although pharmacological inhibition of MYCN is difficult, AURKA inhibitors (E.g., alisertib, CD532) can be used to indirectly target MYCN (38, 41, 42). MYCN protein levels decreased significantly in response to the anti-tumor activities of AURKA inhibitor treatment (39). Additionally, a mechanistic link between mutated *TP53* and elevated AURKA levels has been described (43). Collectively these data point to MYCN as a central mediator of cellular plasticity during NE differentiation.

### 2.4 FOXA1 and FOXA2

Changes in the expression of the Forkhead Box A (FOXA) family of proteins, especially FOXA1 and FOXA2, have been implicated in NEPC development. The FOXA family is a group of pioneer transcription factors that can access condensed chromatin, facilitating the local binding of other regulatory factors (44). FOXA1 expression has been shown to be transiently increased in localized PCa but is lowered following progression to CRPC (45, 46) and further decreased in NEPC (47). Loss of *FOXA1* promotes NE differentiation by upregulating CXCL8 and CXCL8-activated MAPK/ERK phosphorylation. Moreover, FOXA1 is reported to play a reprogramming role in lineage conversion (48), with *FOXA1* mutations altering its pioneering function and perturbing normal luminal epithelial cell differentiation programs (49). FOXA1 interacts with the TET1 protein to mediate local DNA demethylation and histone 3 lysine 4 methylation (50). On the other hand, FOXA2 overexpression has been reported in genetically engineered mouse models of NEPC, a result consistent with clinical NEPC specimens (51, 52). However, FOXA2 expression is not detected in prostate adenocarcinoma samples (51). FOXA2 induces prostate adenocarcinoma-to-NE lineage transition through cooperation with HIF1 and REST (53, 54), and *FOXA2* knockdown reverses this process. Interestingly, FOXA2 has been shown to positively regulate KIT expression in NEPC (55), a receptor tyrosine kinase that is positively correlated with NEPC score and SYP, ENO2, and EZH2 expression (55, 56). Inhibiting the KIT pathway resulted in NEPC growth suppression in both human and mouse NEPC, suggesting the potential utility of targeting KIT in the treatment of NEPC (55). Nevertheless, a better understanding of the roles FOXA1/FOXA2 hold in regulating lineage plasticity and their crosstalk with epigenetic modulators is still key to discover new therapeutic strategies.

## 3 Epigenetic dysregulation affecting lineage plasticity in PCa

Epigenetic modifications (e.g., DNA methylation, histone modification, and chromatin remodeling) are critical regulators of transcriptional states, are known drivers of carcinogenesis, and are tightly associated with NEPC lineage plasticity. Despite similarities in their genomic profiles, NEPC and prostate adenocarcinoma have markedly distinct epigenetic landscapes (4, 37, 57). Notably, AR signaling loss is strongly associated with epigenetic reprogramming and is essential for maintaining luminal cell differentiation in the prostate (58). Moreover, significant evidence demonstrates the importance of epigenetics in the transition to a NEPC phenotype. For instance, epigenetic dysregulation in NEPC has been reported in pathways involving cell-cell adhesion, development, epithelial-mesenchymal transition (EMT), and stem cell regulation and maintenance (4).

### 3.1 DNA methylation

DNA methylation is the most widely studied epigenetic modification and has implications in carcinogenesis, therapy resistance, and lineage plasticity. DNA methylation refers to the covalent attachment of a methyl group to DNA cytosine residues, forming 5-methylcytosine (5mC). *De novo* DNA methylation is catalyzed by the DNA methyltransferases (DNMT) DNMT3a and DNMT3b, while maintenance DNA methylation is catalyzed by DNMT1 (59). Inversely, DNA demethylation is mediated by ten eleven translocation (TET) enzymes. DNA methylation primarily occurs at CpG-rich regions called ‘CpG islands.’ Notably, CpG islands are preferentially located in the 5’ UTR of genes and make up approximately 60% of human gene promoters. Methylation of gene promoters predominantly results in gene silencing by either preventing or promoting the recruitment of regulatory elements (60). Global DNA hypomethylation is frequently observed in cancer and can lead to genomic instability and proto-oncogene activation (60). DNA hypomethylation is postulated to occur early in the development of many types of cancer, driving carcinogenesis. However, PCa breaks away from this common view, instead occurring late in disease progression and likely is involved in the formation and propagation of metastases (61). Site-specific CpG island hypermethylation leading to aberrant gene silencing is frequently observed in PCa (60, 62). DNA methylation is known to disrupt pathways involved in DNA damage repair, hormonal response, tumor-cell invasion and metastasis, and cell cycle control (63, 64). Additionally, DNA methylation can regulate lineage plasticity and therapy resistance through promoter hypermethylation of *CRIP1*, *FLNC*, *RASGRF2*, *RUNX2*, and *HS3ST2*. These genes are important for lineage commitment and differentiation and have been associated with tumor recurrence (65–68). As such, DNA methylation plays a significant role in driving NE transdifferentiation from an adenocarcinoma. For instance, in NEPC samples, *ASXL3* and *SPDEF* genes were found to be hypermethylated, while the NEPC marker *INSM1* and the plasticity gene *CDH2* were hypomethylated (69).

DNA methylation has also been implicated in regulating AR signaling. Specifically, DNA hypermethylation of the *AR* promoter, driven by *PTEN, RB1, and TP53* loss, can partially explain the inactivation of canonical AR signaling in NEPC (70, 71). For instance, in AR-null cell line models and patient tumor samples, DNA methylation has been implicated in regulating AR expression (73–75). Treatment with AR-negative cell lines, DuPro, TSU-PR1, and DU145, with the DNMT inhibitor 5-azacitidine resulted in AR re-expression and concomitant demethylation of the *AR* promoter (73, 75). DNMTs can also regulate AR signaling in a methylation independent manner. Prostate epithelial cells lacking *RB1* demonstrate increased DNMT1 expression. RB1 negatively regulates the transcription factor E2F1, which can activate DNMT1 expression by interacting with the *DNMT1* promoter. Overexpression of E2F1 was sufficient to repress AR expression and *AR* promoter-driven reporter genes (71). Similarly, DNMT1 knockdown renewed AR expression in AR-negative human prostate epithelial cells. DNMT1 directed ChIP analysis showed association of DNMT1 with the AR promoter; however, a lack of *de novo* methylation at the *AR* minimal promoter suggests that DNMT1 represses AR expression via a methylation-independent mechanism (72). These data present DNMT inhibitors as a promising class of drugs for the treatment of AR-null PCa.

### 3.2 Post translational histone modification

Significant evidence has revealed that post-translational histone modifications mediate numerous biological processes in cancer via alterations in chromatin conformation. These modifications include methylation, acetylation, phosphorylation, and ubiquitination; however, histone-related gene expression regulation is predominately regulated by histone methylation and acetylation. Histone acetylation is driven by histone acetyltransferases (HATs) and is deacetylated by histone deacetylases (HDACs). Similarly, histone methylation is catalyzed by histone methyltransferases (KMTs) and reversed by histone demethylases (KDMs). Histone acetylation is commonly associated with transcriptional activation, resulting from alterations in histone net charge. This leads to an open chromatin conformation that is more accessible to transcription factor binding and the regulation of gene transcription. Unlike histone acetylation, methylation does not exclusively lead to translational activation but is instead associated with either gene activation or repression.

#### 3.2.1 Histone methylation and demethylation

AR expression loss has been linked to the repression of histone methylation. Enrichment of repressive histone modifications H3K27me3 and H3K9me2 has been observed in AR-null NEPC PDXs (76). Enhancer of Zeste Homologue 2 (EZH2), the catalytic subunit of the Polycomb Repressive Complex 2 (PRC2), is a master regulator of epigenetic rewiring in NEPC and is the KMT responsible for H3K27me3 marks. Upregulation of EZH2 is a well-established feature of NEPC (4, 37, 39, 77). Genes repressed by EZH2 are also downregulated in tumors from NEPC patients, with EZH2 inhibition reactivating AR signaling, decreasing NE target gene expression, and re-sensitizing tumors to enzalutamide (34). Mechanistically, EZH2 cooperates with lineage-guiding transcription factors to epigenetically regulate gene expression and lineage specificity. Notably, EZH2 has been shown to cooperate with MYCN to suppress AR signaling (39). EZH2 has also been shown to synergize with other epigenetic modifiers, regulating chromatin conformation to promote lineage plasticity. For instance, EZH2 has been linked to DNMT activity, potentially marking genes for *de novo* DNA methylation via a scaffolding mechanism mediated by the long ncRNA HOTAIR (78–80). Similarly, HOTAIR can scaffold EZH2 and lysine-specific demethylase 1 (LSD1), a H3K4 and H3K9 demethylase. EZH2:LSD1 coordinates to repress developmental genes, promoting a cellular state of elevated plasticity (81).

Several studies report that EZH2 mediates NE differentiation. For example, EZH2 can be activated by transcription factor 4 (TCF4), a key transcription factor involved in Wnt/-catenin signaling. Elevated Wnt signaling is a common feature in NEPC, and TCF4 inhibition has been shown to preclude the transdifferentiation of adenocarcinoma to NEPC, following ADT (82). Furthermore, EZH2 activity has been tied to cAMP-response element binding protein (CREB) activation. Inhibition of EZH2 can prevent CREB-induced H3K27 methylation and NE differentiation (83). Finally, MEK-ERK signaling has been shown to regulate EZH2 transcriptional upregulation and recruitment to the promoter of E-cadherin. Therefore, MEK-ERK signaling can facilitate epithelial-mesenchymal plasticity via EZH2 regulation (84, 85). These studies demonstrate EZH2’s role as a master regulator of NEPC lineage plasticity, and its inhibition is a promising strategy for novel NEPC treatment.

In addition to histone methylation, histone demethylation also plays a significant role in regulating NEPC. LSD1 is known to demethylate H3K4 and lysine residues on several non-histone proteins, including, TP53, E2F1, DNMT1, and HIF-1. Notably, LSD1 is an important regulator of AR transcriptional activity, suppressing the transcription of AR target genes (86). Indirect demethylation of H3K9 on AR target genes and cell cycle genes can also be facilitated by LSD1 (87). Interactions between LSD1 and another master epigenetic regulator SRRM4 can produce a neuronal-specific isoform of LSD1, called LSD1 + 8a, that is exclusively expressed in NEPC as opposed to adenocarcinoma. SRRM4 is overexpressed in NEPC and is a powerful driver of transdifferentiation in NEPC (88). This splice variant of LSD1 may act as a biomarker for NEPC and contribute to lineage plasticity in NEPC (28). These epigenetic factors may provide novel targets for NEPC treatment.

#### 3.2.2 Histone acetylation, deacetylation, and chromatin remodeling

Recent studies have shown that the use of modern ARPIs can result in widespread changes in chromatin structure and contribute to lineage plasticity. Histone acetylation and deacetylation are two factors that affect chromatin accessibility (89–92). Notably, the HDAC SIRT1 is upregulated in NEPC, and its overexpression has been shown to promote lineage plasticity (93). The precise mechanism by which SIRT1 promotes lineage plasticity is unknown; however, in breast cancer (94), liver cancer stem cells (95), and bone marrow-derived mesenchymal stem cells (96), SIRT has been shown to promote upregulation of SOX2. SIRT1 mediated SOX2 upregulation may be an avenue whereby SIRT1 promotes lineage plasticity in NEPC, though evidence remains preliminary and evolving.

Other chromatin remodelers in PCa can affect lineage plasticity. *CDH1* loss, for example, has been shown to promote neuronal differentiation and enzalutamide resistance (92). *CDH1* loss resulted in global changes in chromatin accessibility, notably, increasing accessibility for four transcription factors associated with non-luminal lineage program activation. Deletion of each transcription factor by CRISPR-Cas9 knockout re-sensitized cells to enzalutamide (97). The restrictive element-1 silencing transcription factor (REST) cooperates with AR and corepressors such as EZH2 and LSD1 to suppress neuronal differentiation (98, 99). REST silencing attenuated AR signaling and increased NE lineage markers (100). Moreover, loss of REST activity via a splicing-in event by SRRM3/4 can promote expression of BAF53B rather than BAF53A, subunits of the chromatin remodelers, neuron specific BAF (nBAF) complex and canonical BAF complex respectively (12). Exchanging the subunits BAF53A and BAF45A for BAF53B and BAF45B in the nBAF complex has been shown to promote a chromatin switch to a differentiated neuronal phenotype in post-mitotic neurons (101). Moreover, BAF53B and BAF45B are both highly expressed in NEPC and absent in adenocarcinoma (90). Lastly, it has been shown that BET bromodomain proteins are important in NEPC. Specifically, the BET bromodomain protein BRD4, which recognizes H3K27 acetylation, can cooperate with E2F1 to induce lineage plasticity. BET bromodomain inhibition suppressed the induction of lineage plasticity driving genes and suppressed the growth of treatment emergent NEPC cells high in E2F1 (89). All together, these data demonstrate the importance of epigenetic factors in driving lineage plasticity toward a NEPC phenotype.

## 4 Early therapeutic resistance in localized PCa

### 4.1 Early hormone therapy resistance

As previously described, early hormone therapy failure often results in AR-dependent CRPC. Several mechanisms promote the progression to AR-dependent CRPC, including increased AR amplification and mutation, AR-Vs expression, and intra-tumoral steroid hormone synthesis (8).

Increased AR expression allows PCa to circumvent low androgen levels by hypersensitizing tumor cells to androgens, promoting disease progression (102). Several factors can promote increased AR expression, including gene amplification, increased translation, and decreased degradation (8). Moreover, AR expression can be enhanced by increased protein stability via the E3 ligase MID1 (103). AR amplification has been identified in a significant proportion of CRPC cell lines, ranging from 30-80% (104, 105). Several point mutations in the AR gene have been identified that can result in increased AR activity, despite low androgen levels. For instance, mutations in the AR ligand binding domain can decrease ligand specificity, allowing alternate steroid hormones, such as -DHT, progesterone, or DHEA, to activate AR signaling (106). Notably, other mutations that confer resistance to second generation AR agonists, such as F876L, have been identified (107).

Several alternative AR-Vs that confer ADT resistance have been identified. These splice variants often emerge in late stages of PCa, are constitutively active due to the loss of the C-terminus ligand binding domain, and often correlate with a poor prognosis (108, 109). Several common AR-Vs are ARv1, ARv7, and ARv567, with ARv7 being the most well studied (110, 111). ARv7 is constitutively active because it lacks the canonical AR ligand binding site (111). Moreover, ARv7 can heterodimerize with AR to translocate to the nucleus where it acts as a transcription factor (112). ARv7 has been shown to regulate both AR-regulated genes as well as a unique set of AR-independent genes (111). Given the correlation between AR splice variant expression and poor prognosis, the targeting of AR variants may offer novel CRPC treatment with the ARv7 inhibitor niclosamide being recently tested (113).

Another key mechanism contributing to AR-dependent CRPC is the intra tumoral synthesis of androgen, including testosterone and dihydrotestosterone (DHT), which originate from adrenal precursor steroids such as dehydroepiandrosterone (DHEA) (114, 115). Several enzymes and genes involved in intra tumoral androgen synthesis have been identified as potential targets for diagnosis and treatment. One such target is 3β-hydroxysteroid dehydrogenase isoenzyme-1 (3βHSD1, encoded by HSD3B1), which catalyzes the rate-limiting step in the metabolic conversion from DHEA to testosterone and DHT in the adrenal gland. A specific germline missense-encoding variant of HSD3B1(1245A>C) leads to a divergence of enzyme level and downstream androgen synthesis. HSD3B1(1245A) is known as an adrenal-restrictive allele as it codes for an enzyme that is degraded more rapidly, while HSD3B1(1245C), an adrenal-permissive allele, codes for a stable enzyme resistant to proteasomal degradation that promotes robust conversion from DHEA to DHT (116–118). Numerous studies have shown that prostate cancer patients carrying the HSD3B1(1245C) variant are more likely to become resistant to ADT and progress to CRPC and have worse survival outcomes. However, to date there is no known disparity of overall survival time related to HSD3B1.

The upregulation of glucocorticoid receptors (GR) is one AR-independent mechanism known to mediate ADT resistance in PCa. GRs are a part of the nuclear receptor superfamily of transcription factors (119). Like AR, GR undergoes a conformational change in the cytoplasm and is translocated to the nucleus after binding with androgens or glucocorticoids. Once inside the nucleus, GR can bind to androgen responsive elements and regulate the expression of target genes (120). Notably, AR and GR bind to nearly identical DNA motifs (121). One implication of this finding is that amplified GR signaling may be redirected to circumvent AR loss of function under castrate conditions (18). Notably, GR regulates a significant number of genes that are AR pathway-specific (122), and GR activity has been found to replace AR activity in CRPC, following enzalutamide treatment (123, 124).

### 4.2 Radioresistance in PCa

Radiotherapy is a cornerstone of curative PCa treatment, and its combination with ADT has been shown to significantly increase patients’ overall survival (125). Nevertheless, approximately 30-50% of high-risk, clinically localized PCa cases recur within five years of curative intent radiotherapy. While the majority of these recurrences are out of field metastases, PSMA PET scans are shedding increasing light on non-trivial local recurrence rates and offer an opportunity for deeper study of mechanisms driving resistance to radiotherapy/hormone therapy combinations (126). The intra-tumoral genetic and epigenetic heterogeneity observed in PCa has been theorized as a source of resistant subpopulations (127). Moreover, the selective pressure applied by RT may trigger evolutionary adaptations resulting in acquired radioresistance promoted by epigenetic and transcriptional reprogramming (128). Both acquired and intrinsic radioresistance involves changes in biological mechanisms controlling DNA repair, hypoxia response, cell proliferation and survival, EMT, apoptosis inhibition, and autophagy (129). As described below, extensive experimental evidence also supports the role of an aberrant epigenome in driving a radioresistant phenotype.

#### 4.2.1 Alternative AR signaling driving PCa radioresistance

One inference that can be drawn from the significant clinical benefit observed with ADT and RT combinations is that their anti-neoplastic effects are at least additive and more likely synergistic. It has been demonstrated that AR signaling promotes radioresistance by upregulating the transcription of DNA double strand break (DSB) repair genes. Treatment with second-generation anti-androgens was shown to downregulate DNA repair genes in a CRPC model. Moreover, primary PCa was observed to displays a wide spectrum of AR transcriptional output that correlates with the expression of a set of DNA repair genes. The addition of androgens to ionizing radiation (IR) treated PCa cells enhanced DNA damage repair and treatment with anti-androgens increased DNA damage and decreased clonogenic survival (130). Similarly, increased AR expression and activity was observed following RT in several human PCa models, both *in vitro* and *in vivo*. AR expression was also correlated with survival *in vitro* and time to tumor progression *in vivo*. Finally, AR pathway upregulation was found in nearly 20% of patients following RT (131). Taken together, these data demonstrate the strong synergistic effect of ADT and RT. This underscores the important role of AR signaling in therapeutic resistance to genotoxic treatments.

Given the association between AR signaling and radioresistance in localized hormone sensitive disease, it follows that castrate-resistance is similarly tied to radiation response. A possible pathway leading to radioresistance in CRPC is the emergence of AR-Vs. One study has shown that AR-Vs can promote non-homologous end joining (NHEJ) repair when canonical AR signaling is blocked by ARPI (132). Similarly, others have used CRISPR-engineered PCa cell lines to show that AR-Vs can desensitize cells to IR when canonical AR signaling is disturbed (133). ARv7 is the most clinically relevant of the AR-Vs and was shown to significantly promote the DNA damage response (DDR) of PCa cells following severe DNA damage. ARv7 expression was sufficient to upregulate both homologous recombination (HR) and NHEJ in PCa by forming a positive regulatory loop with poly ADP-ribose polymerase 1 (PARP-1) (17). Interestingly, epigenetics seem to play a role in ARv7 expression in PCa via the inclusion of a cryptic exon. The KDM Jumonji domain containing (JMJD) 1A, an eraser of H3K9 methylation, can complex with the splicing factor hnRPNF. This complex binds to methylated H3K9 regions of the cryptic exon located in the intronic sequence between exons 3 and 4, favoring the inclusion of the cryptic exon and ARv7 production. Furthermore, JMJD1A knockdown has been shown to reduce ARv7 levels (134). With AR splice variants promoting radioresistance, inhibiting their expression may re-sensitize tumors to IR.

#### 4.2.2 Epigenetic aberrations promote PCa radioresistance

Several epigenetic events affecting the DDR and cell cycle that can promote tumor radioresistance. Key proteins, such as ataxia-telangiectasia mutated (ATM), DNA dependent protein kinases, -H2AX, breast cancer gene 1/2 (BRCA 1/2), PARP-1, and RAD51, are important in DDR induction (135). Defective DDR pathways are frequently described as drivers of PCa tumorigenesis, with 15-30% of cases displaying DDR instability (136). Several genes, involved in DNA damage are frequently hypermethylated in PCa, including *GPX3*, *MGMT*, and *ASC* (137). Moreover, *ATM* may be hypermethylated, as its functional loss far exceeds its rate of mutation (138). The methylation of DDR genes frequently results in tumorigenesis due to increased chromosomal instability, but impaired DDR can leave tumors more susceptible to RT. For example, patients with *ATM* and *BRCA1/2* mutations exhibited superior tumor response following RT (139).

Conversely, the methylation of genes involved in cell cycle regulation are more often associated with increased radioresistance. For instance, the cyclin-dependent kinase inhibitors (*CDKs*), *CDKN1A* (p21^Cip1^), *CDKN1B* (p27^Kip1^), and *CDKN2A* (p16^Ink4A^), reprimo (*RPRM*), stratifin (*SFN*), and *CDK1* are all frequently hypermethylated and play various roles in tumor radioresistance (140). The CDKNs and their associated pathways have a variety of complex interactions regulating tumor suppression, DDR activation, cell cycle arrest, and apoptosis control (141). This regulation is accomplished by inhibiting cyclin-dependent kinases, with p21 and p27 inhibiting CDK2 and p16 inhibiting CDK4/6. Moreover, activation of p21 signaling has been found to promote radioresistance (142). Similarly, demethylation of p21 and p53 promoters in nasopharyngeal carcinoma restored and activated p21 and p53 activity, leading to cell cycle arrest and apoptosis, resulting in increased radioresistance (143). Cell phase change, due to decreased expression or loss of p27, has been shown to confer radioresistance in esophageal carcinoma and luminal breast cancer (144, 145). Depletion of p16 in cervical cancer led to increased radioresistance and higher self-renewal capability (146). Moreover, wild-type p16 expression was consistent with radiosensitivity in malignant melanoma cells when compared to homozygous p16 deficient cell lines (147). The tumor suppressor RPRM has previously been implicated in triggering p53-mediated G2 cell cycle arrest following DNA damage (148, 149) and negatively regulates ATM levels (150). Moreover, RPRM loss has been shown to confer radioresistance both *in vitro* and *in vivo*, marking RPRM as a potential target for cancer therapy and radiation protection (149, 150). SFN is known to mediate G2 arrest by preventing the nuclear translocation of the CDC2-cyclin B1 complex in response to DNA damage (151). In contrast to other hypermethylated cell-cycle related genes, the hypermethylation of SFN is not associated with greater radioresistance. Instead, cells that lose SFN frequently undergo mitotic catastrophe following DNA damage (151, 152). Interestingly, one study found the expression of SFN to increase with tumor progression; however, the driving mechanism behind the increased expression has not been investigated (151). Still, this group of cell cycle-regulating genes offers many novel targets for radiation protection, and the transient nature of these epigenetic changes may allow for the re-sensitization of radioresistant tumors. Nevertheless, more data are needed before these findings can be effectively translated into the clinic.

Post-translational histone modification is another class of epigenetic modifications that can affect PCa radioresistance. For instance, EZH2 is frequently upregulated and suppresses transcription via H3K27 methylation (153). In addition to driving lineage plasticity, EHZ2 has several cellular functions pertaining to the DDR, including regulating DDR elements and chromatin conformation following IR-induced damage. IR promotes H3K27 trimethylation by EZH2 around DNA damage sites. The resulting chromatin compaction is necessary for efficient DNA damage repair (154). Additionally, EZH2 can cooperate with BRCA1 to maintain a cancer stem cell signature, promoting radioresistance. Treatment with a global histone methylation inhibitor downregulates BRCA1 and EZH2 expression, inhibiting tumorigenicity and radioresistance (155). Similarly, in glioblastoma multiforme cells, EZH2 inhibition has been shown to significantly reduce H3K27 methylation and increased residual H2AX foci following IR. This significantly increased cell cycle arrest at the G2 checkpoint and apoptotic cell death (156). Finally, EZH2 inhibition has been shown to robustly downregulate DDR genes by preventing EZH2-mediated FOXA1 direct methylation (157). Another histone modifier that is frequently overexpressed in PCa is the histone methyltransferase KMT2D. KMT2D serves as an oncogene, promoting tumor growth and metastasis; however, it also has functions regarding radioresistance. Silencing of KMT2D has been shown to promote ROS-mediated DNA damage, leading to apoptosis and senescence. Moreover, KMT2D loss reduced enhancer activity markers H3K4me1 and H3K27ac, which blocked the binding of FOXO3, an important mediator of the cellular oxidative stress response, resulting in suppressed antioxidant gene expression (158). Additionally, KMT2D overexpression can also epigenetically activate PI3K/Akt and upregulate EMT and oncogenic pathways, promoting tumor radioresistance (159).

The PI3K/Akt/mTOR pathway can affect radioresistance in other ways. The Ras family of GTPases plays a key role in various basic cellular functions such as controlling cell proliferation, differentiation, and apoptosis, with Ras stimulations having a wide range of downstream signaling pathways. Phosphoinositide 3-kinases (PI3Ks) are one of the best characterized Ras effector groups, and their activation leads to the induction of the PI3K/Akt/mTOR pathway that affects cell growth, cell cycle, and cell survival (160). Uncontrolled activation of this signaling pathway has been associated with the development of cancer and tumor radioresistance. Radioresistant cell lines have demonstrated an increased activation in the PI3K/Akt/mTOR pathway (161). Moreover, dual inhibition of PI3K and mTOR has resulted in a significant reduction in radioresistance, increasing apoptosis, arrest of the G2/M phase, increased double strand breaks, and reduced cell cycle checkpoint inactivation and autophagy both *in-vitro* and *in-vivo* (162, 163). DNA methylation can promote PI3K/Akt/mTOR pathway-driven radioresistance via the hypermethylation of negative regulators of this pathway (164, 165). Moreover, H3 and H4 acetylation have been shown to activate Akt/mTOR, resulting in increased PCa progression and therapy resistance (166).

## 5 Therapeutic implications

With lineage plasticity driven CRPC posing a challenge for clinical management, novel therapeutic options targeting NEPC are needed. Several clinical trials focusing on agents that target NEPC have been reported, with one of the earliest Phase 2 trials testing the AURKA inhibitor alisertib. Investigators tested alisertib in NEPC patients as AURKA appears to be important in stabilizing MYCN (40). It should be noted that this trial did not require MYCN or AURKA upregulation for enrollment. Although this study did not meet its primary end point of 6-months progression free survival, the subpopulation of patients that exhibited increased AURKA expression (16%) did appear to have longer overall survival (167). Another NEPC targeting agents is rovalpituzumab tesirine, a DLL3-targeted antibody-drug conjugate. This agent was tested in a Phase 1/2 trial that included 18 patients with NEPC, and a 10% objective response rate was observed (168). A comprehensive list of therapies targeting NEPC from 2017 to 2023 can be found in **Table 1**.

**Table 1:** Clinical trials of non-epigenetic therapies targeting neuroendocrine prostate cancer (2017-2023).

	Drug	Target	Combination Agent(s)	Phase	Status	Trial Identifier
**Targeted/Immuno-therapy**	Apalutamide, Cetrelimab	AR Inhibitor, anti-PD-1		2	R	NCT04926181
	Tarlatamab	DLL3/CD3 (Bispecific T-Cell Engager)		1	R	NCT04702737
	HPN328	anti-DLL3/anti-CD3/anti-albumin (T-Cell Engager)		1/2	R	NCT04471727
	PT217	DLL3/CD47 (Bispecific Antibody)		1	NYR	NCT05652686
	BXCL701, Pembrolizumab	DPP8/DPP9 Inhibitor, anti-PD-L1		1/2	R	NCT03910660
	Niraparib, Cetrelimab	PARP Inhibitor, anti-PD-1	Carboplatin, Cabazitaxel	2	R	NCT04592237
	Cabozantinib S-malate, Ipilimumab, Nivolumab	TK Inhibitor, anti-CTLA4, anti-PD-1		2	R	NCT03866382
	Regorafenib, Tislelizumab	VEGFR1-3/TIE2/PDGFR-β/FGFR/KIT/RET/RAF Inhibitor, anti-PD-1		2	NYR	NCT05582031
	Lenvatinib, Pembrolizumab	VEGFR2 Inhibitor, anti-PD-L1		2	R	NCT04848337
	Tivozanib, Atezolizumab	VEGFR/TK Inhibitor, anti-PD-L1		1/2	R	NCT05000294
	Lenvatinib, Pembrolizumab	VEGFR1-3 Inhibitor, anti-PD-L1		1/2	R	*NCT02861573
	Vibostolimab-Pembrolizumab	anti-TIGIT/anti-PD-L1		1/2	R	*NCT02861573
**Immuno-therapy**	Pembrolizumab	anti-PD-L1	Carboplatin, Cabazitaxel	2	NYR	NCT05563558
	Pembrolizumab	anti-PD-L1	Carboplatin, Cisplatin, Docetaxel, Etoposide	1	R	NCT03582475
	Avelumab	anti-PD-L1		2	C	NCT03179410
	Nivolumab, Ipilimumab	anti-PD-1, anti-CTLA4		2	R	NCT03333616
	XmAb20717	anti-PD-1/anti-CTLA4		1	C	NCT03517488
	Nivolumab, Ipilimumab	anti-PD-1, anti-CTLA4	Carboplatin, Cabazitaxel	2	R	NCT04709276
	PDR001, LAG525	anti-PD-1, anti-LAG-3		2	C	NCT03365791
**Targeted**	Berzosertib	ATR Inhibitor	Topotecan Hydrochloride	2	ANR	NCT03896503
	Abiraterone, Apalutamide, Leuprolide	CYP17A1 Inhibitor, AR Inhibitor, GnRH Agonist		2	ANR	NCT03902951
	Erdafitinib	FGFR Inhibitor		2	R	NCT04754425
	Goserelin	GnRH Agonist	Docetaxel, Prednisode	2	R	*NCT03696186
	Goserelin	GnRH Agonist	Carboplatin, Doxetaxel, Prednisone	2	R	*NCT03696186
	[225]-FPI-2059	NTSR1 (Targeted Alpha Therapy)	[111In]-FPI-2058	1	R	NCT05605522
	Olaparib	PARP inhibitor	Carboplatin, Cabazitaxel, Prednisone	2	ANR	NCT03263650
	ORIC-944	PRC2 Inhibitor		1	R	NCT05413421

NEPC, Neuroendocrine Prostate Cancer; ANR, Active Not Recruiting; C, Completed; NYR, Not Yet Recruiting; R, Recruiting. *: Clinical trial with >1 intervention targeting NEPC.

In addition to NEPC targeted therapies, drugs that target the NEPC epigenome and delay or reverse NE differentiation are promising. EZH2 is the most well studied epigenetic factor that is dysregulated in NEPC (39, 77). Preclinical NEPC models treated with EZH2 inhibitors have demonstrated attenuation of MYCN driven NEPC phenotypes and re-sensitization to ARPI treatment (39, 169). For instance, the EZH2 inhibitor, PF-06821497, is currently being tested in a Phase 1 study in metastatic CRPC patients (NCT03460977). Similarly, ORIC-944 is being tested in a Phase 1/1b trial in patients with metastatic CRPC (NCT05413421).

BET bromodomain proteins also offer a potential route for epigenetic targeting of NEPC. Preclinically, treatment with BRD4 inhibitors, either in monotherapy or in combination with ARPIs, have exhibited anti-tumor properties in PCa (170–172). Early investigation into the BET inhibitor ZEN-3694 has found that patients with NEPC tumors that exhibit high E2F1/BRD4 activity may be more susceptible to BET inhibition (89, 173). ZEN-3694 is now being tested in combination with enzalutamide and pembrolizumab in a Phase II trial, comprised of treatment emergent NEPC patients (NCT04471974). Similarly, the combination of ZEN-3694 and enzalutamide vs. enzalutamide alone is being tested in patients whose disease responded poorly to abiraterone, with the hope that this will enrich the cohort for patients with tumors that have undergone lineage plasticity (NCT04986423). A comprehensive overview of the current clinical trials for epigenetic drugs targeting NEPC can be found in **Table 2**.

**Table 2:** Clinical trials of epigenetic therapies targeting neuroendocrine prostate cancer.

	Drug	Target	Combination Agent(s)	Indication	Phase	Status	Trial Identifier
**Epigenetic**	JBI-802	LSD1/HDAC6/HDAC8 Inhibitor		NEPC	1/2	Recruiting	NCT05268666
	CC-90011	LSD1 Inhibitor	Itraconazole, Rifampicin	NEPC	1	Active, not recruiting	NCT02875223
**Epigenetic/Targeted**	EPZ-6438, Abiraterone	EZH2 Inhibitor, CYP17A1 Inhibitor	Prednisone	(*Phase 1b Intervention 1*) SCNEPC, NEPC, mPC	1/2	Recruiting	*NCT04179864
	EPZ-6438, Enzalutamide	EZH2 Inhibitor, AR Inhibitor		(*Phase 1b Intervention 2*) SCNEPC, NEPC, mPC	1/2	Recruiting	*NCT04179864
**Epigenetic/Targeted/Immuno-therapy**	ZEN-3694, Enzalutamide, Pembrolizumab	BET Inhibitor, AR Inhibitor, anti-PD-L1		Transdifferentiated mCRPC, t-SCNEPC, SCNEPC	2	Recruiting	NCT04471974

NEPC, Neuroendocrine Prostate Cancer; mPC, Metastatic Prostate Cancer; SCNEPC, Small-Cell Neuroendocrine Prostate Cancer; t-SCNEPC, Treatment-Emergent Small-Cell Neuroendocrine Prostate Cancer. *: Same clinical trial, different interventions.

Other potential avenues for epigenetic targeting are LSD1 and the DNMTs. LSD1 is a KDM that is overexpressed in androgen-independent PCa and modulates FOXA1-dependent, AR-associated lineage plasticity and stem cell-associated gene expression (174, 175). Several clinical studies testing LSD1 inhibition have been proposed but were later terminated for various reasons (NCT02712905, NCT02217709, and NCT01253642); however, recruitment for Phase 1/2 testing of JBI-802, an LSD1/HDAC6 inhibitor, is currently ongoing (NCT05268666). Preclinical testing of DNMT inhibitors have shown promising results, with DNMT inhibition re-sensitizing resistant NE-like cell lines to ARPIs (176, 177). Notably, the DNMT inhibitors decitabine and azacytidine already have FDA approval for the treatment of myelodysplastic syndromes and could be repurposed for the treatment of NEPC. However, Phase 2 testing of DNMT inhibitors in CRPC did not show strong anti-tumor activity (178, 179).

Drugs that inhibit lineage plasticity by targeting the epigenome may also be used as radiosensitizers in the future. For instance, one pre-clinical study showed that EZH2 inhibition dramatically enhanced CRPC cells to genotoxic stress, demonstrating the potential utility in EZH2 inhibitors in sensitizing cancers that overexpress EZH2-activated DDR genes to genotoxic agents (157). Moreover, BRD4 is essential for the repair of DNA DSBs by mediating NHEJ, and BRD4 is negatively associated with outcome following RT (180). LSD1 also offers a potential option for radiosensitizing PCa, as pre-clinical data has shown that LSD1 knockdown can significantly enhance radiosensitivity (181). Finally, DNMT inhibitors and HDAC inhibitors have also shown radiosensitizing effects (182, 183). However, evidence supporting the efficacy of epigenetic targeting in radiosensitizing PCa has not progressed beyond pre-clinical investigation to date.

Despite the potential of new epigenetic and NE-targeting therapies in NEPC treatment, their development and clinical deployment has proven challenging. One major hurdle for many novel epigenetic therapies is their lack of specificity, often affecting a wide range of transcriptional networks leading to undesired off-target results and side-effects (10). Nevertheless, epigenetic targeting in NEPC offers a promising therapeutic avenue with potential to bring new options to the clinic.

## 6 Conclusion

Prostate cancer encompasses a range of natural histories, spanning from indolent disease to treatment-refractory CRPC/NEPC. Therapy-induced NEPC is marked by lineage plasticity, loss of luminal markers, and acquisition of NE features, such as SYP and CHGA. While genetic mutations, such as loss of PTEN, TP53, and RB1, and MYCN amplification have been implicated in this lineage transition, they are insufficient to induce the transdifferentiation of adenocarcinoma to NEPC. Instead, increasing evidence points to an aberrant epigenome as a major driver of lineage plasticity. Hyper-methylation of the AR promoter may partially explain the loss of canonical AR signaling observed in NEPC. Similarly, abnormal function of master epigenetic regulators, such as EZH2, promote NE lineage programs. Epigenetic dysregulation can also affect PCa radioresistance by altering the expression of genes involved in DDR and cell cycle. NEPC’s reliance on epigenetic machinery has provided a new avenue for treating a disease that, so far, has been nearly incurable. Epigenome-targeting drugs have shown promise for treating NEPC in preclinical and early-clinical studies, and similar mechanisms are beginning to be exploited to re-sensitize tumors to RT. Despite their potential, numerous challenges remain before these epigenetic therapies can be successfully implemented in modern treatment regimens for NEPC. In summary, understanding the mechanisms that promote lineage plasticity, from tumorigenesis to the emergence of treatment resistance, is imperative in contemporary prostate cancer research and represents a critical step in the discovery of new targets and more effective therapies to prevent and treat lethal PCa.

## Author contributions

JI and SGa did the literature review and wrote the initial draft, made the figure, and worked on the revisions. AM constructed the tables. RL wrote the abstract. JN, CEW, CW, and SGu provided valuable suggestions for the manuscript. OM reviewed the final version and approved the final version for submission. All authors contributed to the article and approved the submitted version.
